# The effectiveness of citrates and pyridoxine in the treatment of kidney stones

**DOI:** 10.25122/jml-2023-0234

**Published:** 2023-06

**Authors:** Mihai Andrei Ene, Petrişor Aurelian Geavlete, Cătălina Elena Simeanu, Cătălin Andrei Bulai, Cosmin Victor Ene, Bogdan Florin Geavlete

**Affiliations:** 1 Department of Urology, Sf. Ioan Emergency Clinical Hospital, Bucharest, Romania; 2 Department of Medicine, Carol Davila University of Medicine and Pharmacy, Bucharest, Romania; 3 Department of General Surgery, Sf. Ioan Emergency Clinical Hospital, Bucharest, Romania

**Keywords:** citrates, kidney stones, pyridoxine, calcium

## Abstract

The prevalence of nephrolithiasis is increasing across all demographic groups. Apart from the morbidity associated with an acute occurrence, preventative treatment is essential for stone disease, which can become a long-term problem. Simple interventions like fluid intake optimization and dietary modification are effective for most stone types. However, patients with specific metabolic abnormalities may require pharmaceutical therapy if lifestyle changes are insufficient to reduce the risk of stone recurrence. The treatment of citrates and/or pyridoxines may help eliminate or prevent recurrences of kidney stones, especially when they are composed of uric acid, calcium oxalate, calcium phosphate, or the latter two together. In cases of struvite stones, which often necessitate a surgical approach, acetohydroxamic acid emerges as a valuable second-line treatment option. Thiol-binding agents may be needed for cystinuria, as well as lifestyle modifications. Successful treatment reduces stone recurrence and the need to remove stones surgically.

## INTRODUCTION

Approximately 5 to 15% of the population worldwide suffer from kidney stones, without a clear pattern and with an increasing trend in recent years [1, 2]. In the United States, kidney stones are experienced by at least 1 in 11 individuals during their lifetime [3]. Moreover, there are variations in susceptibility based on race and sex, with white men exhibiting a higher risk compared to Asian women. The incidence of the disease peaks between the third and fourth decade of life and is less prevalent before the age of 20 [4].

Over the past three decades, the lifetime prevalence of kidney stone disease has increased to around 14%, affecting several developed countries [5]. This upward trend can be attributed to multiple factors, including changes in diet and lifestyle, rising rates of obesity [6] and diabetes, urbanization due to migration from rural areas, and the effects of global warming, which can lead to elevated calcium concentrations in the urine, thereby promoting stone formation [7].

The etiology of kidney stones is multifactorial, influenced by genetic and environmental factors. It is clear that inheritance plays a role in monogenic diseases such as cystinuria, Dent's disease, and primary hyperoxaluria [8], but also in idiopathic stone formation [9], although the genes involved are unknown. Diet plays a key role in expressing the tendency to form stones [9, 10].

There is a high incidence of kidney stones among people with chronic kidney disease [11, 12]. Additionally, all the silent features of the metabolic syndrome, such as type 2 diabetes, increased body mass index (BMI), hypertension, and dyslipidemia, have been identified as independent contributors to kidney stone formation [13]. Moreover, there is a significant correlation between stone formers and several risk factors for coronary heart disease, including smoking, hypertension, hypercholesterolemia, and obesity [14].

In adults, calcium oxalate stones are more common than calcium phosphate stones. Typically, the initial nidus of most stones comprises calcium oxalate monohydrate (whewellite) or dihydrate (weddelite), often supplemented by calcium phosphate (CaP). About 5% to 15% of the stones are uric acid and struvite, while less than 1% are cystine, ammonia-magnesium phosphates, protease inhibitor, 2,8-dihydroxyadenine (2,8-DHA), and xanthine [15]. Occasionally, insoluble drugs like ephedrine, triamterene, or indinavir can also contribute to stone formation [16]. Understanding the specific causes of each stone type is crucial, as some are linked to dietary and environmental factors, while others are associated with specific pathologies (Table 1).

**Table 1:** Causes of different stone types

Pathologies that cause primarily calcium oxalate stones	Pathologies that cause primarily calcium phosphate stones	Pathologies that cause either calcium oxalate or calcium phosphate stones	Pathologies that cause uric acid stones	Pathologies that cause cystine stones	Pathologies that cause struvite stones
**Hyperoxaluria**	**Hypercalciuria with normocalcemia and metabolic acidosis**	**Hypocitraturia**	**Low urine pH**	**Inherited gene defects in cystine transport**	**Urinary tract infection with urea-splitting organisms**
Primary hyperoxaluria – Type 1, Type 2	Distal renal tubular acidosis	Secondary to metabolic acidosis	Gouty diathesis	SLC3A1 (Type I or type A cystinuria)	Proteus mirabilis
Enteric hyperoxaluria: - Bariatric surgery - Fat malabsorption from any cause - Small bowel resection		Secondary to hypokalemia	Diabetes	SLC7A9 (Type non-I or type B cystinuria)	Proteus sp., Providencia, Enterobacter, Bordetella, Bacteroides, Staph aureus, Corynebacterium, Ureaplasma
Dietary hyperoxaluria: - Excess vitamin C - Low calcium diet		Idiopathic	Obesity		Serratia, Pseudomonas, Klebsiella, Aeromonas, Pasteurella (occasionally)
**Hyperuricosuria**		**Hypercalciuria with normocalcemia**	Idiopathic (metabolic syndrome)		
High purine diet		Idiopathic	Bowel disease (especially colon resection)		
Myeloproliferative disorder		Granulomatous diseases (sarcoid)	**Low urine volume**		
**Low urine volume that persists**		**Hypercalciuria with hypercalcemia**	**Hyperuricosuria**		
Diarrheal states		Primary hyperparathyroidism	High protein diet		
		Granulomatous diseases (sarcoid)	Uricosuric drugs		
		Malignancy (rare)	Myeloproliferative disorders		
		Vitamin D excess	Phosphoribosyl pyrophosphate synthetase superactivity		
		Hyperthyroidism	Hypoxanthine– guanine phosphoribosyl transferase deficiency		

The formation of a kidney stone occurs when the concentration of salts in the urine surpasses the saturation point, which is influenced by the pH in the urine. Uric acid and cystine stones are more likely to form in acidic urine (low pH), whereas calcium phosphate stones are more likely to form in alkaline urine (high pH) [17].

When patients are diagnosed with urinary stones, several techniques can be used for rapid results at low risk. Minimally invasive surgical techniques continue to evolve, offering innovative approaches tailored to different pathologies, enabling personalized treatment options [18]. It is important to keep in mind the fact that a successful treatment reduces stone recurrence and the need to remove stones surgically.

## CALCIUM OXALATE STONES PREVENTION

Hypercalciuria, hyperoxaluria, hyperuricosuria, hypocitraturia, and low urine volume are important urinary risk factors associated with stones made of calcium oxalate [19]. Therefore, calcium stones can be prevented by reducing urinary calcium and oxalate concentrations, increasing urinary inhibitor levels, such as citrate, and increasing urine volume [20].

Among the metabolic causes of hypercalciuria are primary hyperparathyroidism and chronic acidemia. The treatment of primary hyperparathyroidism patients with hypercalciuria is parathyroidectomy [21]. Chronic metabolic acidosis can lead to hypercalciuria by promoting calcium loss from bones and hypocitraturia through increased active proximal citrate absorption. Potassium citrate is an effective solution for preventing stones in such patients. Also, potassium bicarbonate can be mentioned as an alternative [22].

An individual with hyperoxaluria has an excretion of oxalate in the urine greater than 45 mg per day. There is no clear optimal cutoff point for hypercalciuria, nor one for urinary oxalate excretion. Oxalate excretion can increase the risk of stone formation even at levels above 25 mg/day, which falls within the normal range [17, 23].

Increased oxalate intake can lead to higher urinary oxalate excretion, thereby increasing the likelihood of nephrolithiasis. Following urine measurements, a reduction in oxalate excretion indicates the need to limit dietary oxalate intake as a preventive measure against stone formation [17, 24].

As a coenzyme of alanine-glyoxylate aminotransferase (AGT), pyridoxine (vitamin B6) increases the conversion of glyoxylate to glycine instead of oxalate, being used in treating primary hypercalcemia [17, 25]. However, there is currently no randomized controlled trial that has assessed the effectiveness of pyridoxine in preventing stones in individuals with idiopathic hyperoxaluria. Utilizing pyridoxine at doses between 10 and 500 mg/day is thought to reduce urinary oxalate or the recurrence rate of stones [26-28] in patients who produce oxalate calcium stones [28, 29] in uncontrolled studies. According to Curhan *et al*., women who consume high amounts of pyridoxine are less likely to develop stones than men [30].

Enteric hyperoxaluria, characterized by the binding of 90% of dietary oxalate to calcium in the small intestine, results in 90% of oxalate excretion in the stool [17]. Furthermore, 10% is absorbed in the colon and excreted in the urine. Hyperoxaluria is commonly associated with digestive disorders like short gut syndrome, bowel inflammation, and bariatric surgery. In these cases, excess fat binds to dietary calcium, leading to increased intestinal absorption of free oxalate [31]. Treatment involves increasing hydration and taking calcium supplements, such as citrate or carbonate, to decrease intestinal oxalate absorption, leading to oxalate precipitation in the intestinal lumen. It is recommended that 1 to 4 grams be taken with meals in divided doses three to four times daily. Patients with kidney stones prefer calcium citrate over calcium carbonate because calcium citrate is more soluble and more effective in the presence of achlorhydria [17, 32].

In primary hyperoxaluria, a genetic defect in glyoxylate biosynthesis leads to oxalate overproduction and urinary oxalate excretion exceeding 135 to 270 mg/day [17]. There are three types of primary hyperoxaluria, the first accounting for 90% of cases. In type 1, there is a reduction in the activity of hepatic peroxisomal alanine: glyoxylate aminotransferase AGT [33, 34]. Treatment for primary hyperoxaluria includes consuming enough liquid daily to produce 3 liters of urine, along with the use of potassium citrate (0.15 mg/kg), oral phosphate supplements (30–40 mg/kg of orthophosphate), and magnesium oxide (500 mg/day/m2) to prevent calcium oxalate precipitation [17, 35, 36]. For patients with primary hyperoxaluria of type 1, pyridoxine is prescribed at a starting dose of 5 mg/kg (which can be titrated up to 20 mg/kg if no response is observed) [17]. Pyridoxine is effective in about 50% of patients with type 1, and a 3- to 6-month trial is recommended [25].

An individual with hyperuricosuria excretes more than 800 mg of uric acid per day in men and more than 750 mg per day in women [37]. This excessive uric acid excretion is often associated with a high-protein diet for patients with calcium oxalate stones. Hyperuricosuria is believed to decrease the solubility of calcium oxalate, thereby contributing to stone formation [38]. It is important to note that patients with hyperuricosuric calcium stones have a higher urinary pH as well as a higher uric acid level in their urine [39, 40]. In a randomized controlled trial, calcium stone recurrences among these patients were reduced by allopurinol, while lowering protein intake was also found to be beneficial [41].

Hypocitraturia is another significant risk factor for kidney stone formation. Adults with this pathology usually excrete less than 320 mg/day of citrate [17]. In the case of citraturia, a reading below 100 mg/day is classified as severe, and a reading between 100 and 320 mg/day is classified as moderate-severe [42]. A hypocitraturia patient is 16-63% more likely to form kidney stones containing calcium [43, 44]. Kidney stones with hypocitraturia are characterized by calcium oxalate and calcium phosphate crystallization and nucleation. The presence of potassium citrate can impede this step [45].

Various alkaline citrates are available for treating hypocitraturia, with potassium citrate being preferred over sodium citrate due to its ability to increase urinary calcium excretion [46]. Calcium oxalate is lowered below the saturation point by citrate binding to calcium, preventing calcium crystals from forming [47]. In response to enhanced citrate clearance, potassium citrate metabolism produces high alkalinity. This process leads to increased citrate concentration and pH in the urine. The rise in dissociated calcium anions reduces the activity of calcium ions while simultaneously decreasing oxalate saturation in the urine [45]. Thus, as noted by Prezioso *et al*. in the chapter “Dietary treatment of hypocitraturia", the medicinal use of alkaline citrate salts is recommended in treating kidney stones with hypocitraturia and preventing their recurrence [2].

## CALCIUM PHOSPHATE STONES PREVENTION

Calcium phosphate stones form in alkaline urine (usually urine pH>6.0), usually as the result of distal renal tubular acidosis [17]. Most patients with calcium phosphate stones do not have metabolic acidosis, and their persistently alkaline urine pH is not understood [48]. The presence of stones composed of calcium phosphate correlates with a more serious kidney condition [49, 50]. As with calcium oxalate stones, prevention measures are similar.

However, alkali therapy should be used cautiously because it affects urinary pH and precipitates calcium phosphate crystals [51]. Managing patients with calcium phosphate stones and concurrent hypocitraturia can be challenging. While alkali administration may help correct hypocitraturia in such cases, it may also lead to higher urinary pH levels, increasing the risk of calcium phosphate stone formation. It is recommended to stop alkali therapy when urine pH rises above 6.5 without significant changes in urine citrate or urine calcium excretion [17].

## URIC ACID STONE PREVENTION

Uric acid stones are associated with various conditions, including metabolic syndrome [52], diabetes mellitus [53, 54], gout, chronic diarrhea illness [31], obesity [55, 56], and malignancies that increase tissue turnover and uric acid production [17]. In adults, the formation of uric acid stones often results from a reduction in urine acid solubility due to a low urinary pH rather than hyperuricosuria, making high urine acidity a major risk factor. Uric acid stone formers typically have a defect in ammonia excretion at baseline and in response to an acid load [57, 58]. In this sense, uric acid stone formation may be a manifestation of metabolic syndrome [58].

Metabolic syndrome and diabetes mellitus lead to decreased ammonia production, resulting in a lower urinary pH, which promotes the formation of uric acid stones [17]. Additionally, chronic diarrhea can cause a loss of bicarbonate, further increasing urine acidity. Similar to gout, continuous acidic urine can lead to the formation of uric acid stones, often resulting from impaired ammonium excretion [59]. Ammonia synthesis is stimulated by insulin in individuals without diabetes. In patients with recurrent uric acid stones who are not diabetic [58], insulin resistance is associated with a tendency for acid urine excretion [52, 60]. In low urinary pH conditions, uric acid can precipitate into stones even at excretion rates of 600 to 800 mg/day and urinary volumes of 1 to 1.5 liters [17, 61].

Management of uric acid stone formation typically involves potassium citrate, which is recommended at doses of 60-80 meq/day [62]. Adequate fluid intake also helps solubilize uric acid. Increasing urine pH to 6–6.5 can reduce urinary supersaturation with uric acid and minimize stone recurrence [58]. However, in individuals with diabetes, serum potassium levels should be closely monitored to avoid hyperkalemia. Dietary protein restriction is recommended if urine uric acid excretion is elevated [58].

## CYSTINE STONE PREVENTION

Patients with inherited defects in amino acid transport in the kidneys and intestines tend to develop cystine stones [58]. Due to defective nephron reabsorption, cystine is excreted in the urine more frequently. Cystine's limited solubility can lead to stone formation. Timely initiation of preventive therapy is crucial upon diagnosis, as cystine stones have the potential to grow very large and tend to recur frequently. When a patient has cystinuria, their renal function decreases from an early age [63], and their kidney pathology shows diffuse interstitial fibrosis and collecting duct plugging [58, 64].

Diagnosing cystinuria can be achieved by examining the patient's family history, analyzing stones, or measuring urine cystine excretion. A healthy person excretes about 30 mg of cystine per day, while a person with cystine stones excretes about 400 mg per day [58]. The solubility of cysteine increases in alkaline urine, although at urine pH over 7, it can still range between 175 to 360 mg/L. To achieve an average cystine concentration below 243 mg/L (a desirable goal), high fluid intake is prescribed, both at bedtime and throughout the day, based on the known cystine excretion rates [58]. By adding potassium alkali at a rate of 10-20 meq/tid, urine pH can be raised if it falls below 7 [65].

## STRUVITE STONE PREVENTION

Stones containing magnesium ammonium phosphate, known as struvite stones, are caused by bacteria that split urea, like *Klebsiella, Proteus, Pseudomonas*, and enterococci. Hydrolysis of urea releases hydroxyl ions, resulting in alkaline urine that promotes the formation of struvite stones [17]. Known for their rapid growth and staghorn formation, these stones are challenging to treat and usually require expert urological intervention [38, 68]. When complete stone removal is not feasible due to complicating factors, the use of urease inhibitors has been shown to reduce the growth rate of struvite stones. Three randomized controlled trials have demonstrated that acetohydroxamic acid decreases stone growth in this scenario [69-72]. There are side effects associated with the use of this medication, including headaches, thrombophlebitis, tremors, nausea, and vomiting. There is no indication of citrate therapy for this kind of stone.

## CONCLUSION

Managing recurrent stone disease requires both lifestyle and pharmacological interventions. Treatment decisions should consider the type of stone and metabolic evaluation, as well as any underlying comorbid condition. Considering that all stones are caused by urinary supersaturation with stone matter, the goal is to reduce or eliminate this condition. Implementing preventive measures following the first stone episode is crucial to prevent recurrence, reduce costs, and minimize risks. In addition to fluid intake, alkaline citrate salts and/or pyridoxine can be useful in treating this condition. Given the increasing prevalence of stone diseases in the population, it is necessary to advance our understanding of their pathogenesis and expand our therapeutic options.
